# Predictive Value of Pro-BNP for Heart Failure Readmission after an Acute Coronary Syndrome

**DOI:** 10.3390/jcm10081653

**Published:** 2021-04-13

**Authors:** Alberto Cordero, Elías Martínez Rey-Rañal, María J. Moreno, David Escribano, José Moreno-Arribas, Maria A. Quintanilla, Pilar Zuazola, Julio Núñez, Vicente Bertomeu-González

**Affiliations:** 1 Cardiology Department, Hospital Universitario de San Juan, 03550 Alicante, Spain; eliasmartinezreyranal@gmail.com (E.M.R.-R.); mjmoga6@gmail.com (M.J.M.); d.escribanoalarcon@gmail.com (D.E.); jomoreno@gmail.com (J.M.-A.); mariamteati@hotmail.com (M.A.Q.); pilarzuazola@gmail.com (P.Z.); vbertog@gmail.com (V.B.-G.); 2 Unidad de Investigación en Cardiología, Fundación para el Fomento de la Investigación Sanitaria y Biomédica de la Comunitat Valenciana (FISABIO), 46020 Valencia, Spain; 3 Centro de Investigación Biomédica en Red de Enfermedades Cardiovasculares (CIBERCV CB16/11/00226-CB16/11/00420), 28028 Madrid, Spain; yulnunez@gmail.com; 4 Cardiology Department, Hospital Clínico Universitario, 46010 Valencia, Spain; 5 Instituto de Investigación Sanitaria (INCLIVA), Hospital Clínico Universitario de Valencia, 46010 Valencia, Spain

**Keywords:** pro-BNP, heart failure, acute coronary syndrome

## Abstract

Background: N-terminal pro-brain natural peptide (NT-pro-BNP) is a well-established biomarker of tissue congestion and has prognostic value in patients with heart failure (HF). Nonetheless, there is scarce evidence on its predictive capacity for HF re-admission after an acute coronary syndrome (ACS). We performed a prospective, single-center study in all patients discharged after an ACS. HF re-admission was analyzed by competing risk regression, taking all-cause mortality as a competing event. Results are presented as sub-hazard ratios (sHR). Recurrent hospitalizations were tested by negative binomial regression, and results are presented as incidence risk ratio (IRR). Results: Of the 2133 included patients, 528 (24.8%) had HF during the ACS hospitalization, and their pro-BNP levels were higher (3220 pg/mL vs. 684.2 pg/mL; *p* < 0.001). In-hospital mortality was 2.9%, and pro-BNP was similarly higher in these patients. Increased pro-BNP levels were correlated to increased risk of HF or death during the hospitalization. Over follow-up (median 38 months) 243 (11.7%) patients had at least one hospital readmission for HF and 151 (7.1%) had more than one. Complete revascularization had a preventive effect on HF readmission, whereas several other variables were associated with higher risk. Pro-BNP was independently associated with HF admission (sHR: 1.47) and readmission (IRR: 1.45) at any age. Significant interactions were found for the predictive value of pro-BNP in women, diabetes, renal dysfunction, STEMI and patients without troponin elevation. Conclusions: In-hospital determination of pro-BNP is an independent predictor of HF readmission after an ACS.

## 1. Introduction

Coronary heart disease is the leading risk factor for heart failure incidence [1,2,3], and acute coronary syndrome (ACS) is the most frequent and threatening clinical presentation. The falling of in-hospital and short-term mortality of ACS patients has shifted the impact of the disease, increasing the percentage of patients with chronic coronary heart disease at risk of heart failure (HF) readmissions [4,5,6].

In-hospital [4] and post-discharge [7,8] HF have a large influence on ACS prognosis and, consequently, individual estimation of the actual risk of HF might be very relevant. Several biomarkers have proven to be crucial for the diagnosis, treatment and prognosis assessment in patients with HF [9,10,11]. Among these, natriuretic peptides have some of the most convincing evidence supporting its role in HF diagnosis and congestion [11,12,13]. Pro-BNP serum levels have a recognized predictive value for mortality and major cardiovascular events in ACS patients [14,15,16,17]. The aim of our study was to assess the predictive value of pro-BNP for HF incidence in patients discharged after an ACS.

## 2. Materials and Methods

We designed a retrospective study of all consecutive patients admitted for an ACS between December 2012 and March 2018 in Hospital Universitario de San Juan, Alicante (Spain), based on the ongoing ACS registry of the Cardiology Department. The diagnosis of ACS was defined by (1) typical clinical symptoms of chest pain; (2) electrocardiographic changes indicative of myocardial ischemia/lesion; and/or (3) elevation of serum markers of myocardial damage. Pro-BNP is routinely measured in all patients in the first blood sample obtained after an overnight fasting, usually within 24 h of admission. Risk stratification was performed using the GRACE score [18], and patients with scores of more than 140 were considered as high-risk. The primary endpoint assessed through follow-up was HF re-admission, registered as a hospital admission whose main diagnosis was congestive HF according to clinical guidelines [19]. The study design and results presentation were made according to the STROBE (strengthening the reporting of observational studies in epidemiology) [20] recommendations (Table S1). The ethics committee of the institution has approved the protocol and informed consent of the registry.

Demographic characteristics of the patients, risk factors for coronary artery disease (smoking, hypertension, dyslipidemia and diabetes mellitus), medical history, laboratory data during the hospitalization, vital signs on admission, treatment and diagnosis at discharge were collected from all patients using the electronic database of our institution. History of heart failure was codified if patients had at least one hospitalization with such diagnosis at discharge or the typical signs and symptoms of heart failure and a compatible echocardiogram. Patients underwent an echocardiography within 48 h of admission, and the left ventricular ejection fraction (LVEF) was calculated using Simpson’s method. After overnight fasting, a blood sample was obtained for biochemical determinations. The glomerular filtration rate (GFR) was estimated from serum creatinine values with the CKD-EPI equation [21]; GFR of less than 60 mL/min/m^2^ was considered indicative of renal dysfunction. Hypertension, dyslipidemia and diabetes mellitus were considered to be present in patients receiving specific therapy for these diseases. We recorded a history of coronary heart disease if patients had a previous diagnosis of myocardial infarction, stable or unstable angina, or angina-driven coronary revascularization. Completeness of revascularization was prospectively determined after the procedure, on the basis of the intended equivalent anatomic revascularization using segment numbering of vessels with a diameter of more than 1.5 mm [22]. Comorbidity was assessed by the Charlson index, adapted for patients with coronary heart disease [23]. All diagnoses and medical histories were obtained by the cardiologist in charge of the database. All clinical variables were recorded at the time of discharge from the hospital. After discharge, patient follow-up was performed by means of telephone calls, revision of clinical reports and revision of electronic medical records, in order to obtain clinical status and outcome events. All primary care visits, medical interventions, emergency calls, visits to the emergency room and hospital readmissions are recorded in a centralized electronic medical records system. The ongoing registry of our institution has a straightforward methodology and data collection protocol. Current investigation is based on widely available measures and, therefore, missing data were very scarce.

Quantitative variables with normal distribution are presented as means (standard deviation, SD), and differences were assessed by ANOVA. Variables with non-normal distribution, like pro-BNP, are presented as medians (interquartile range, IQR), and differences were analyzed using the Kolmogorov-Smirnov test. Qualitative variables are presented as percentages and were compared between groups using the Student t and Chi-squared tests. Prior to entry into regression models, raw pro-BNP values were natural log-transformed and then expanded using fractional polynomials so as not to assume linearity of effect. In-hospital mortality predictors were assessed by logistic regression. Variables with more than 10% of missing data would be imputed to the mean.

The incidence of post-discharge HF could be affected by patient’s death, so the usual techniques for time-to-event analysis would provide biased or un-interpretable results due to the presence of competing risks, and the Kaplan-Meier method would overestimate real HF incidence [22,24]. To avoid such effects, we applied the model introduced by Fine and Gray [25] to test competing events. Regression models were adjusted by all the variables that obtained significant differences in the univariate analysis (patients who had HF readmission vs. no HF readmissions) and also those variables that could have a clinically relevant implication. The incidence of HF is presented in cumuled incidence function graphs, and results of the multivariate analysis, performed by competing risk regression, as sub-hazard ratio (sHR) and corresponding 95% confidence intervals (CIs). Therefore, sHR represents the hazard ratio of HF taking all-cause mortality as a competing event. Post-estimation assessment was based on the model’s discrimination using Harrell’s C-statistic. The analysis of recurrent cardiovascular events was performed by negative binomial regression, and results are presented as incidence rate ratio (IRR) and 95% Cis [26]. Models were accurately calibrated (Harrels C-statistic 0.83, 95% CI 0.80–0.85; *p* < 0.001). Patients lost to follow-up were categorized as missing, as were those who lacked any of the main variables for the analyses, although these were less than 5%. Statistical significance was accepted at *p* < 0.05. All analyses were performed using STATA 14.3 (StataCorp. 2009. Stata Statistical Software: Release 14. College Station, TX, USA: StataCorp LP).

## 3. Results

We included 2133 patients (Table 1) and 528 (24.8%) had HF within the ACS hospitalization, and they had higher pro-BNP than the rest: 3220 pg/mL vs. 684.2 pg/mL (*p* < 0.001).

### 3.1. In-Hospital Mortality

**Figure 1:**
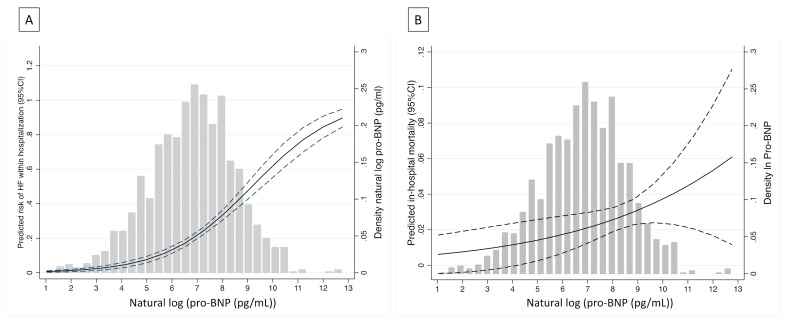
Histograms for pro-BNP distribution (density) and predicted risk of in-hospital heart failure (**A**) or death (**B**).

In-hospital mortality was 2.9% (61 patients) and pro-BNP was higher in patients who died: 9522.0 pg/mL vs. 949.1 pg/mL (*p* < 0.001). As shown in Figure 1, pro-BNP values were correlated with a gradual increase in the risk of HF or in-hospital death.

### 3.2. Post-Discharge Heart Failure Admissions

Post-discharge follow-up was available in 94% of the cohort, with a median follow-up of 38 months (IQR 26–48). Of these patients, 292 (14.1%) died, most of them (*n* = 206, 9.9% of the total) from cardiovascular causes. Moreover, 243 (11.7%) had at least one hospital readmission for HF, and 151 (7.1%) had more than one. There were relevant differences in clinical features and medical treatments at discharge between patients that did or did not have a hospital readmission for HF (Table 2); differences were taken into consideration in the multivariate analysis. After adjustment for age, gender, risk factors, previous cardiovascular disease, LVEF, medical treatments, hemoglobin and GFR, the competing risk regression showed the protective effect of complete revascularization on HF readmission, whereas several variables were associated with higher risk of HF (Table 3). Pro-BNP was independently associated with first and recurrent HF re-admissions.

**Figure 2:**
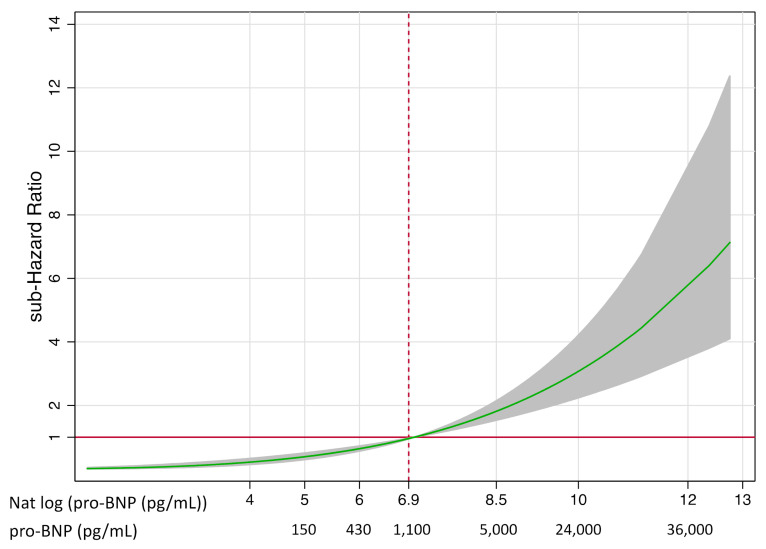
Relative sub-hazard risk of readmission for heart failure according to pro-BNP levels.

**Figure 3:**
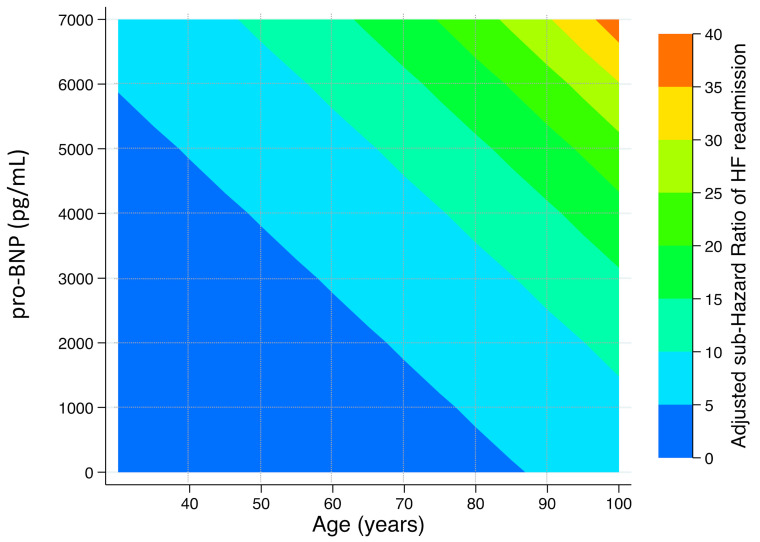
Relative sub-hazard risk of readmission for heart failure according to age and pro-BNP levels.

The risk of HF readmission increased with pro-BNP values (Figure 2). In order to have clinically relevant and accessible tool, we finally designed a risk matrix to represent the risk of HF readmission according to age and serum levels of pro-BNP. The risk of HF readmission at any given age and pro-BNP values is presented in Figure 3.

We finally performed several subgroup analyses. As shown in Table 4, a positive interaction was found for female gender first HF readmission; the predictive value of pro-BNP was similar according to DM, previous HF or previous HF. In contrast, the predictive value of pro-BNP for recurrent HF readmission was higher for patients with GFR > 60 mL/min/1.72 m^2^**,** non-STEMI or those without DM or without troponin elevation.

## 4. Discussion

The results of this large cohort study in ACS patients demonstrate the predictive value of pro-BNP for HF readmission after an ACS. There was a gradual increase of HF readmission with higher pro-BNP values, and the risk cumulatively increased with age. Since clinical features and event rates are similar to previous reports [1,2,3,4,5,6,7,8,9,10,11,14,15,16,17,26,27,28], we believe that our results are reasonably representative of daily clinical practice.

The incidence of HF has increased over the last decades, with large social, demographic, economic and health implications [1,2,3]. Therefore, all strategies directed to predict and prevent its incidence are highly relevant. Since coronary heart disease is the leading risk factor for HF [3], we analyzed the effect of a well-established biomarker of congestion and HF, measured in one of the most critical moments for the myocardium: an ACS. HF within the acute phase of an ACS impairs in-hospital and post-discharge prognosis [4,29], and post-discharge HF quadruples the risk of death [7]. Thus, predicting HF incidence after an ACS is clinically relevant and, not surprisingly, several clinical variables, and scores, have even proposed to define patients’ individual risk of HF re-admission [21,30]. Our results highlight the predictive role of pro-BNP in a large cohort of ACS patients. We found a strong relationship between pro-BNP and in-hospital HF or mortality, which might help identify patients with poorer outcomes in whom close or intense management should be mandatory.

Natriuretic peptides are cardiac-derived hormones with natriuretic, diuretic, and vasodilatory effects. They are secreted into the circulation in response to increased cardiac wall stress and have robust diagnostic power for cardiac vs. non-cardiac dyspnea as well as prognostic significance in patients with HF in terms of recurrent hospitalizations and death [31]. Pro-BNP has been reported as one of the strongest predictors of death among patients with or without HF [13], especially when determined in an acute clinical setting [32]. Left ventricle remodeling after an ACS precludes HF onset, although recent data with cardiac magnetic resonance have shown that deterioration of the ejection fraction and myocardial damage are the main determinants of poor prognosis rather than just left ventricle enlargement [33]. Our results are also in line with these findings and might reflect the fact that pro-BNP elevation already reveals more extensive myocardial damage or might precede myocardial fibrosis and remodeling [34]. In fact, our risk matrix showed that at any given age or LVEF pro-BNP is an independent predictor of HF. Patients who develop HF within the ACS hospitalization are at higher risk of post-discharge HF, regardless of LVEF [4]; the fact that patients with elevated pro-BNP are also at higher risk of HF might help to identify patients with a neurohormal activation similar to clinical HF [9,10]. This might also explain why cardiac natriuretic peptides are one of the best biomarkers for predicting outcomes in ACS patients [14,15,16,17] and, moreover, should be determined routinely in all ACS patients.

We also found relevant interactions between pro-BNP and female gender, diabetes, renal dysfunction, STEMI or troponin elevation. Competing risk regression is the most accurate statistical approach for heart failure incidence since all-cause mortality is a competing event [24]. All these variables are clearly associated to higher mortality rates and the presence of significant interactions reflects a modification on the predictive value of pro-BNP; thereafter, pro-BNP determinations could be even more relevant in these clinical subgroups. Moreover, women, diabetes and renal dysfunction are known to have higher prevalence of un-diagnosed HF [19] and, maybe, pro-BNP could be unmasking some of these cases.

We believe that our results support the use of pro-BNP in daily clinical practice for better characterizing the actual prognosis in patients after ACS, although the implications for medical treatment are less clear. Myocardial damage [35] and inflammation dysregulation [36] following an ACS promote left ventricular remodeling and loss of function. Mineral corticoid receptor antagonist reduced major cardiovascular events and mortality in patients with myocardial infarction and left ventricle dysfunction in the EPHESUS Trial [37]; nonetheless, a lack of benefit on HF readmissions after an ACS has been reported in real-world patients [38]. Future applications with early initiation of therapies such as sacubitril/valsartan, currently being tested in the PARADISE-MI trial [39], or sodium-glucose co-transporter-2 inhibitors [40] could change the landscape in HF prevention after an ACS.

Our study has several limitations that deserve further consideration. First, this is a single-center study, and results might not be representative of all clinical settings. Second, there may be many unmeasured confounders or details about physician or patient decision-making that are not captured in our data collection. The analyses used observational, non-randomized data, so associations between variables and outcomes may be distorted by unmeasured confounders. Furthermore, there may have been appropriate contraindications to adjunctive pharmacotherapy or invasive angiography that were not collected. Third, patients who died before a blood sample could be obtained were not included in the study, so it was not possible to assess the role of pro-BNP in those very-high-risk patients. Similarly, troponin values are routinely determined in all patients, mainly in the first hours of admission for patients’ rule-in or rule-out. Nonetheless, there is a wide variability in subsequent determinations and, therefore, the maximal values are not systemically available, and patients are classified by the presence of troponin elevation or not. Finally, long-term outcomes could be subject to different circumstances outside the scope of our center’s follow-up protocol. Since clinical features and outcomes were similar to previous reports [1,2,3,4,5,6,7,8,9,10,11,14,15,16,17,26,27,28], we believe that our results are reasonably representative of current practice.

## 5. Conclusions

In conclusion, in-hospital determination of pro-BNP has an independent predictive value for HF readmission after an ACS. At any given age, LVEF, or other clinical variables, pro-BNP levels are correlated with risk of HF readmission after hospital discharge. These results support the use of pro-BNP in daily clinical practice for the better characterization of actual risk of HF in patients with ACS. The implications for medical treatment still need to be elucidated.

## Figures and Tables

**Table 1:** Overall characteristics of the cohort.

N	2133
Age	68.5 (12.7)
Male gender	74.3%
Diabetes	34.9%
Hypertension	66.5%
Current smokers	30.9%
Dyslipidemia	51.0%
Previous coronary heart disease	24.6%
Previous heart failure	3.2%
Previous stroke	6.7%
Peripheral arterial disease	7.8%
Atrial fibrillation	8.3%
Chronic obstructive pulmonary disease	9.7%
ST-elevation ACS	36.7%
GRACE score	145.8 (41.8)
GRACE > 140	50.6%
Charlson index	2.4 (2.1)
Charlson index ≥ 4	21.4%
Left ventricle ejection fraction (%)	54.2 (11.9)
Angiography	95.1%
Revascularization	89.3%
Hemoglobin (gL/dL)	13.3 (4.9)
Creatinine (mg/dL)	1.1 (0.5)
Glomerular filtration rate (mL/min/1.72 m^2^)	73.1 (23.6)
Total cholesterol (mg/dL)	159.4 (63.6)
LDL cholesterol (mg/dL)	92.1 (37.7)
HDL cholesterol (mg/dL)	39.6 (13.1)
HbA1c (%)	6.4 (1.3)
NT pro-BNP (pg/mL)	977.3 (295.4–2923)

HDL: high-density lipoprotein; LDL: low-density lipoprotein.

**Table 2:** Clinical features and medical treatments at discharge of the cohort according to whether they had any hospital readmission for heart failure or not.

	Heart Failure Readmission		
	No	Yes	*p*
*N*	1829 (88.3%)	243 (11.7%)	
Age	67.6 (12.7)	75.8 (10.1)	<0.001
Male gender	75.8%	62.5%	<0.001
Diabetes	32.2%	54.9%	<0.001
Hypertension	64.1%	86.2%	<0.001
Current smokers	32.6%	18.0%	<0.001
Dyslipidemia	50.1%	58.0%	0.02
Previous coronary heart disease	23.5%	33.0%	0.002
Previous heart failure	2.8%	6.3%	0.005
Previous stroke	5.9%	13.0%	<0.01
Peripheral arterial disease	7.2%	12.1%	0.01
Atrial fibrillation	7.5%	14.3%	0.001
Chronic obstructive pulmonary disease	9.4%	12.1%	0.20
ST-elevation acute coronary syndrome	38.2%	25.0%	<0.01
GRACE score	143.3 (42.2)	158.0 (38.6)	<0.001
GRACE >140	48.9%	64.3%	<0.01
Charlson index	2.3 (2.1)	3.2 (2.4)	0.49
Charlson index ≥4	20.0%	32.4%	<0.001
Left ventricle ejection fraction (%)	54.5 (12.7)	49.1 (12.7)	<0.001
Angiography	96.7%	92.2%	0.001
Revascularization	91.6%	84.0%	<0.001
Hemoglobin (gL/dL)	13.5 (5.1)	12.1 (2.9)	<0.001
Creatinine (mg/dL)	1.1 (0.5)	1.3 (0.7)	<0.001
GFR (mL/min/1.72 m^2^)	75.1 (23.0)	57.4 (22.3)	<0.001
Total cholesterol (mg/dL)	160.7 (65.3)	149.2 (48.8)	0.01
LDL cholesterol (mg/dL)	93.1 (38.2)	84.2 (32.8)	<0.001
HDL cholesterol (mg/dL)	39.2 (13.2)	42.4 (12.5)	<0.001
HbA1c (%)	6.4 (1.3)	6.8 (1.5)	<0.001
NT pro-BNP (pg/mL)	802 (254–2208)	3342 (1343–8312)	<0.001
Aspirin	93.3%	87.8%	0.003
Clopidogrel	53.0%	61.3%	0.02
Ticagrelor	17.8%	13.5%	0.11
Prasugrel	15.3%	3.6%	<0.001
ACEI/ARB	81.1%	78.8%	0.43
Beta-blockers	85.2%	84.7%	0.84
Diuretics	19.5%	42.8%	<0.001
Statins	92.7%	86.9%	0.003
Nitrates	8.6%	19.4%	<0.001
Calcium channel blocker	13.5%	20.3%	0.007
Anticoagulants	6.0%	11.2%	0.003
Insulin	8.8%	15.3%	0.002
Oral antidiabetics	24.4%	37.4%	<0.001
SGLT2 inhibitors	0.5%	1.2%	0.27
Mineral corticoid receptor antagonist	9.9%	17.1%	0.001
Proton pump inhibitors	76.0%	71.2%	0.11

ACEI: angiotensin-converter enzyme inhibitors; ARB: angiotensin receptor blocker; HDL: high-density lipoprotein; LDL: low-density lipoprotein; MRA: mineral corticoid receptor antagonist.

**Table 3:** Independent predictors of heart failure readmission.

	Any HF Readmission Sub-Hazard Ratio (95% CI)	Recurrent Readmissions IRR (95% CI)
Complete revascularization	0.71 (0.53–0.95); *p* = 0.022	0.60 (0.47–0.76); *p* < 0.001
Atrial fibrillation	1.18 (0.80–1.76); *p* = 0.396	1.31 (1.05–1.65); *p* = 0.019
Diabetes mellitus	1.33 (1.02–2.10); *p* = 0.002	1.53 (1.28–1.85); *p* < 0.001
Female gender	1.42 (1.06–1.92); *p* = 0.022	1.20 (1.00–1.45); *p* = 0.049
Pro-BNP (pg/mL)	1.47 (1.31–1.66); *p* < 0.001	1.45 (1.36–1.54); *p* < 0.001
Heart failure within hospitalization	1.50 (1.09–2.77); *p* = 0.040	1.55 (1.10–2.17); *p* = 0.012
Arterial hypertension	1.88 (1.25–2.83); *p* = 0.002	2.22 (1.68–2.94); *p* < 0.001

IRR: incidence rate ratio.

**Table 4:** Subgroup analysis for the predicted value of pro-BNP heart failure readmission predictors.

		Any HF Readmission Sub-Hazar Ratio (95% CI)	Interaction	Recurrent Readmissions IRR (95% CI)	Interaction
Gender	male	1.38 (1.21–1.59); *p* < 0.001	*p* = 0.002	1.31 (1.21–1.43); *p* < 0.001	*p* = 0.152
	female	1.69 (1.41–2.04); *p* < 0.001		1.61 (1.42–1.84); *p* < 0.001	
GFR	>60 mL/min/1.72 m^2^	150 (1.26–1.77); *p* < 0.001	*p* = 0.170	1.50 (1.36–1.66); *p* < 0.001	*p* = 0.015
	<60 mL/min/1.72 m^2^	1.40 (1.18–1.58); *p* < 0.001		1.29 (1.17–1.43); *p* < 0.0.01	
Diabetes	no	1.68 (1.42–2.00); *p* < 0.001	*p* = 0.077	1.57 (1.40–1.74); *p* < 0.001	*p* = 0.003
	yes	1.37 (1.19–1.58); *p* < 0.001		1.34 (1.22–1.47); *p* < 0.001	
Previous HF	no	1.48 (1.32–1.67); *p* < 0.001	*p* = 0.278	1.40 (1.30–1.49); *p* < 0.001	*p* = 0.201
	yes	1.59 (0.88–2.90); *p* = 0.127		1.30 (0.95–1.90); *p* = 0.07	
STEMI	No	1.54 (1.37–1.73); *p* < 0.001	*p* = 0.647	1.53 (1.41–1.66); *p* < 0.001	*p* = 0.003
	Yes	1.46 (1.16–1.85); *p* < 0.001		1.22 (1.08–1.38); *p* < 0.001	
Troponin elevation	No	1.56 (1.31–1.80); *p* < 0.001	*p* = 0.958	1.59 (1.42–1.78); *p* < 0.001	*p* = 0.003
	Yes	1.42 (1.21–1.67); *p* < 0.001		1.30 (1.19–1.41); *p* < 0.001	

HF: Heart failure; IRR: incidence rate ratio.

## Data Availability

Results and database will be available under demand.

## Supplementary Materials

The following are available online at https://www.mdpi.com/article/10.3390/jcm10081653/s1. Table S1: STROBE Statement—Checklist of items that should be included in reports of *cohort studies.*

## References

[1] Fernández-Gassó L., Hernando-Arizaleta L., Palomar-Rodríguez J.A., Abellán-Pérez M.V., Hernández-Vicente Á., Pascual-Figal D.A. (2019). Estudio poblacional de la primera hospitalización por insuficiencia cardiaca y la interacción entre los reingresos y la supervivencia. Rev. Española Cardiol..

[2] Filippatos G., Angermann C.E., Cleland J.G.F., Lam C.S.P., Dahlström U., Dickstein K., Ertl G., Hassanein M., Hart K.W., Lindsell C.J. (2020). Global Differences in Characteristics, Precipitants, and Initial Management of Patients Presenting with Acute Heart Failure. JAMA Cardiol..

[3] Lawson Claire A., Zaccardi F., Squire I., Okhai H., Davies M., Huang W., Mamas M., Lam Carolyn S.P., Khunti K., Kadam Umesh T. (2020). Risk Factors for Heart Failure. Circ. Heart Fail..

[4] Agra Bermejo R., Cordero A., García-Acuña J.M., Gómez Otero I., Varela Román A., Martínez Á., Álvarez Rodríguez L., Abou-Jokh C., Rodríguez-Mañero M., Cid Álvarez B. (2018). Determinants and Prognostic Impact of Heart Failure and Left Ventricular Ejection Fraction in Acute Coronary Syndrome Settings. Rev. Española Cardiol..

[5] Cordero A., Rodriguez-Manero M., Bertomeu-Gonzalez V., Garcia-Acuna J.M., Baluja A., Agra-Bermejo R., Alvarez-Alvarez B., Cid B., Zuazola P., Gonzalez-Juanatey J.R. (2021). Insuficiencia cardiaca de novo tras un síndrome coronario agudo en pacientes sin insuficiencia cardiaca ni disfunción ventricular izquierda. Rev. Española Cardiol..

[6] Agarwal M.A., Fonarow G.C., Ziaeian B. (2021). National Trends in Heart Failure Hospitalizations and Readmissions from 2010 to 2017. JAMA Cardiol..

[7] Taniguchi T., Shiomi H., Morimoto T., Watanabe H., Ono K., Shizuta S., Kato T., Saito N., Kaji S., Ando K. (2017). Incidence and Prognostic Impact of Heart Failure Hospitalization During Follow-Up After Primary Percutaneous Coronary Intervention in ST-Segment Elevation Myocardial Infarction. Am. J. Cardiol..

[8] Wellings J., Kostis J.B., Sargsyan D., Cabrera J., Kostis W.J. (2018). Risk Factors and Trends in Incidence of Heart Failure Following Acute Myocardial Infarction. Am. J. Cardiol..

[9] Nunez J., Llacer P., Bertomeu-Gonzalez V., Bosch M.J., Merlos P., Garcia-Blas S., Montagud V., Bodi V., Bertomeu-Martinez V., Pedrosa V. (2016). Carbohydrate Antigen-125-Guided Therapy in Acute Heart Failure: CHANCE-HF: A Randomized Study. JACC Heart Fail..

[10] Ouwerkerk W., Zwinderman A.H., Ng L.L., Demissei B., Hillege H.L., Zannad F., van Veldhuisen D.J., Samani N.J., Ponikowski P., Metra M. (2018). Biomarker-Guided Versus Guideline-Based Treatment of Patients with Heart Failure: Results from BIOSTAT-CHF. J. Am. Coll. Cardiol..

[11] Nguyen K., Fan W., Bertoni A., Budoff M.J., Defilippi C., Lombardo D., Maisel A., Szklo M., Wong N.D. (2020). N-terminal Pro B-type Natriuretic Peptide and High-sensitivity Cardiac Troponin as Markers for Heart Failure and Cardiovascular Disease Risks According to Glucose Status (from the Multi-Ethnic Study of Atherosclerosis [MESA]). Am. J. Cardiol..

[12] Yancy C.W., Jessup M., Bozkurt B., Butler J., Casey D.E., Colvin M.M., Drazner M.H., Filippatos G.S., Fonarow G.C., Givertz M.M. (2017). 2017 ACC/AHA/HFSA Focused Update of the 2013 ACCF/AHA Guideline for the Management of Heart Failure: A Report of the American College of Cardiology/American Heart Association Task Force on Clinical Practice Guidelines and the Heart Failure Society of America. J. Card. Fail..

[13] York M.K., Gupta D.K., Reynolds C.F., Farber-Eger E., Wells Q.S., Bachmann K.N., Xu M., Harrell F.E., Wang T.J. (2018). B-Type Natriuretic Peptide Levels and Mortality in Patients with and without Heart Failure. J. Am. Coll. Cardiol..

[14] de Lemos J.A., Morrow D.A., Bentley J.H., Omland T., Sabatine M.S., McCabe C.H., Hall C., Cannon C.P., Braunwald E. (2001). The Prognostic Value of B-Type Natriuretic Peptide in Patients with Acute Coronary Syndromes. N. Engl. J. Med..

[15] O’Donoghue M.L., Morrow D.A., Cannon C.P., Jarolim P., Desai N.R., Sherwood M.W., Murphy S.A., Gerszten R.E., Sabatine M.S. (2016). Multimarker Risk Stratification in Patients with Acute Myocardial Infarction. J. Am. Heart Assoc..

[16] Lindholm D., James S.K., Gabrysch K., Himmelmann A., Cannon C.P., Mahaffey K.W., Steg P.G., Held C., Siegbahn A., Wallentin L. (2018). Association of multiple biomarkers with risk of all-cause and cause-specific mortality after acute coronary syndromes: A secondary analysis of the plato biomarker study. JAMA Cardiol..

[17] Zhang D.Q., Li H.W., Chen H.P., Ma Q., Chen H., Xing Y.L., Zhao X.Q. (2018). Combination of Amino-Terminal Pro- BNP, Estimated GFR, and High-Sensitivity CRP for Predicting Cardiorenal Syndrome Type 1 in Acute Myocardial Infarction Patients. J. Am. Heart Assoc..

[18] Granger C.B., Goldberg R.J., Dabbous O., Pieper K.S., Eagle K.A., Cannon C.P., Van de Werf F., Avezum A., Goodman S.G., Flather M.D. (2003). Predictors of hospital mortality in the global registry of acute coronary events. Arch. Internet Med..

[19] Ponikowski P., Voors A.A., Anker S.D., Bueno H., Cleland J.G.F., Coats A.J.S., Falk V., Gonzalez-Juanatey J.R., Harjola V.P., Jankowska E.A. (2016). 2016 ESC Guidelines for the diagnosis and treatment of acute and chronic heart failure: The Task Force for the diagnosis and treatment of acute and chronic heart failure of the European Society of Cardiology (ESC)Developed with the special contribution of the Heart Failure Association (HFA) of the ESC. Eur. Heart J..

[20] von Elm E., Altman D.G., Egger M., Pocock S., Gotzsche P.C., Vandenbroucke J. (2007). The Strengthening the Reporting of Observational Studies in Epidemiology (STROBE) statement: Guidelines for reporting observational studies. PLoS Med..

[21] Levey A.S., Stevens L.A., Schmid C.H., Zhang Y.L., Castr A.F., Feldman H.I., Kusek J.W., Eggers P., Van Lente F., Greene T. (2009). A new equation to estimate glomerular filtration rate. Ann. Internet Med..

[22] Rodriguez-Manero M., Cordero A., Kreidieh O., Garcia-Acuna J.M., Seijas J., Agra-Bermejo R.M., Abou-Jokh C., Alvarez-Rodriguez L., Alvarez-Iglesias D., Lopez-Palop R. (2017). Proposal of a novel clinical score to predict heart failure incidence in long-term survivors of acute coronary syndromes. Int. J. Cardiol..

[23] Sachdev M., Sun J.L., Tsiatis A.A., Nelson C.L., Mark D.B., Jollis J.G. (2004). The prognostic importance of comorbidity for mortality in patients with stable coronary artery disease. J. Am. Coll. Cardiol..

[24] Cordero A., Rodriguez-Manero M., Bertomeu-Gonzalez V., Gonzalez-Juanatey J.R. (2021). Managing NSTEMI in older patients. Lancet.

[25] Fine J.P., Gray R.J. (1999). A Proportional Hazards Model for the Subdistribution of a Competing Risk. J. Am. Stat. Assoc..

[26] Rogers J.K., Pocock S.J., McMurray J.J., Granger C.B., Michelson E.L., Ostergren J., Pfeffer M.A., Solomon S.D., Swedberg K., Yusuf S. (2014). Analysing recurrent hospitalizations in heart failure: A review of statistical methodology, with application to CHARM-Preserved. Eur. J. Heart Fail..

[27] Cordero A., Lopez-Palop R., Carrillo P., Moreno-Arribas J., Bertomeu-Gonzalez V., Frutos A., Garcia-Carrilero M., Gunturiz C., Bertomeu-Martinez V. (2016). Comparison of Long-Term Mortality for Cardiac Diseases in Patients with Versus without Diabetes Mellitus. Am. J. Cardiol..

[28] Cui X., Zhou J., Pivodic A., Dahlström U., Ge J., Fu M. (2020). Temporal trends in cause-specific readmissions and their risk factors in heart failure patients in Sweden. Int. J. Cardiol..

[29] Batchelor R.J., Dinh D., Brennan A., Wong N., Lefkovits J., Reid C., Duffy S.J., Chan W., Cox N., Liew D. (2020). Relation of Timing of Percutaneous Coronary Intervention on Outcomes in Patients with Non-ST Segment Elevation Myocardial Infarction. Am. J. Cardiol..

[30] Hall T.S., von Lueder T.G., Zannad F., Rossignol P., Duarte K., Chouihed T., Solomon S.D., Dickstein K., Atar D., Agewall S. (2019). Left ventricular ejection fraction and adjudicated, cause-specific hospitalizations after myocardial infarction complicated by heart failure or left ventricular dysfunction. Am. Heart J..

[31] Melendo-Viu M., Abu-Assi E., Manzano-Fernández S., Flores-Blanco P.J., Cambronero-Sánchez F., Dobarro Pérez D., Cespón Fernández M., Sánchez Galian M.J., Gómez Molina M., Caneiro-Queija B. (2020). Incidence, prognosis and predictors of heart failure after acute myocardial infarction. REC CardioClinics.

[32] Sionis A. (2016). Comentarios a la guía ESC 2016 sobre el diagnóstico y tratamiento de la insuficiencia cardiaca aguda y crónica. Rev. Española Cardiol..

[33] Bayes-Genis A., Barallat J., Galán A., de Antonio M., Domingo M., Zamora E., Gastelurrutia P., Vila J., Peñafiel J., Gálvez-Montón C. (2015). Estrategia multimarcador para estratificar el pronóstico en insuficiencia cardiaca. Valor de los marcadores neurohumorales: Neprilisina frente a NT-proBNP. Rev. Española Cardiol..

[34] Rodriguez-Palomares J.F., Gavara J., Ferreira-González I., Valente F., Rios C., Rodríguez-García J., Bonanad C., García del Blanco B., Miñana G., Mutuberria M. (2019). Prognostic Value of Initial Left Ventricular Remodeling in Patients with Reperfused STEMI. JACC Cardiovasc. Imaging.

[35] Lindsey M.L., Deleon-Pennell K.Y., Bradshaw A.D., Larue R.A.C., Anderson D.R., Thiele G.M., Baicu C.F., Jones J.A., Menick D.R., Zile M.R. (2020). Focusing Heart Failure Research on Myocardial Fibrosis to Prioritize Translation. J. Card. Fail..

[36] Santos-Gallego C.G., Requena-Ibanez J.A., San Antonio R., Ishikawa K., Watanabe S., Picatoste B., Flores E., Garcia-Ropero A., Sanz J., Hajjar R.J. (2019). Empagliflozin Ameliorates Adverse Left Ventricular Remodeling in Nondiabetic Heart Failure by Enhancing Myocardial Energetics. J. Am. Coll. Cardiol..

[37] Peet C., Ivetic A., Bromage D.I., Shah A.M. (2019). Cardiac monocytes and macrophages after myocardial infarction. Cardiovasc. Res..

[38] Pitt B., Remme W., Zannad F., Neaton J., Martinez F., Roniker B., Bittman R., Hurley S., Kleiman J., Gatlin M. (2003). Eplerenone, a selective aldosterone blocker, in patients with left ventricular dysfunction after myocardial infarction. N. Engl. J. Med..

[39] Agra-Bermejo R., Cordero A., Rodríguez-Mañero M., García Acuña J.M., Álvarez Álvarez B., Martínez Á., Álvarez Rodríguez L., Abou-Jokh C., Cid Álvarez B., González-Juanatey J.R. (2018). Clinical impact of mineralocorticoid receptor antagonists treatment after acute coronary syndrome in the real world, A propensity score matching analysis. Eur. Heart J. Acute Cardiovasc. Care.

[40] Santos-Gallego Carlos G., Requena-Ibanez Juan A., San Antonio R., Garcia-Ropero A., Ishikawa K., Watanabe S., Picatoste B., Vargas-Delgado Ariana P., Flores-Umanzor Eduardo J., Sanz J. (2021). Empagliflozin Ameliorates Diastolic Dysfunction and Left Ventricular Fibrosis/Stiffness in Nondiabetic Heart Failure. JACC Cardiovasc. Imaging.

